# Virtual Reality as an Alternative to Conventional Anatomy Teaching: A Systematic Review of Randomised Controlled Trials

**DOI:** 10.7759/cureus.112410

**Published:** 2026-07-10

**Authors:** Rifat Ershad, Imaad Khan, Syed Gilani, Rawaha Husam Al-Deen

**Affiliations:** 1 Department of General Medicine, Basildon University Hospital, Basildon, GBR

**Keywords:** anatomy education, knowledge retention, medical education, randomised controlled trial, systematic review, virtual reality

## Abstract

Traditional anatomy education, anchored in cadaveric dissection and two-dimensional (2D) resources, faces mounting pressure from reduced curricular hours, educator shortages, and rising costs. Virtual reality (VR) offers an immersive and repeatable alternative; however, its objective efficacy relative to established methods remains debated. The aim of this study was to evaluate the efficacy of VR-based instruction versus conventional teaching in undergraduate anatomy education, with a particular focus on knowledge acquisition, long-term retention, and learner satisfaction. A systematic review was conducted in accordance with Preferred Reporting Items for Systematic Reviews and Meta-Analyses (PRISMA) guidelines. PubMed/MEDLINE (Medical Literature Analysis and Retrieval System Online) and the Cochrane Central Register of Controlled Trials (CENTRAL) were searched from inception to August 2024. Eligibility was restricted to randomised controlled trials (RCTs) involving undergraduate medical students. Risk of bias was assessed using the Cochrane RoB 2.0 tool. Eight RCTs (n ≈ 480 participants) were included. VR interventions consistently matched or exceeded traditional methods in immediate knowledge acquisition, with the most pronounced benefits observed in spatially complex anatomical domains. VR was seen to be non-inferior at five to nine days and eight weeks, and superior at four months (p < 0.001), though the latter finding derives from an add-on design in which both groups received conventional teaching. The non-inferiority of immersive VR versus cadaveric bone models in skeletal anatomy was also seen. Evidence quality was rated moderate overall, with performance bias the primary methodological limitation across studies. VR is an effective educational adjuvant that enhances long-term retention in anatomy education and is supported by emerging biometric evidence of reduced extraneous cognitive load. The evidence supports its integration within a blended learning model rather than as a wholesale replacement for cadaveric dissection. Scalability and cost-effectiveness strengthen the case for its adoption in resource-constrained curricula.

## Introduction and background

Anatomy remains the structural foundation of medical training, underpinning clinical reasoning, diagnostic accuracy, and surgical safety [1]. For centuries, cadaveric dissection has served as the cornerstone for anatomy education, providing students with direct tactile engagement with three-dimensional (3D) structures and demonstrating the biological variation inherent to human specimens. However, the 21st-century undergraduate medical curriculum is under considerable pressure. Across the United Kingdom and internationally, dedicated anatomy teaching hours have been progressively and substantially reduced as integrated curricula have expanded, with cadaveric dissection eliminated entirely from some programmes [2]. Concurrently, a global shortage of clinically trained anatomy educators, driven by the progressive reduction of demonstrator posts and the failure to replace retiring anatomists, together with escalating costs associated with cadaver procurement and laboratory maintenance, has compelled institutions to seek credible pedagogical alternatives [3].

The primary cognitive challenge in anatomy learning, particularly for novice students, lies in spatial visualisation. Learners must mentally translate two-dimensional (2D) diagrams, histological slides, and cross-sectional images into coherent 3D mental models, a process that imposes significant extraneous cognitive load [4]. Students with lower baseline spatial ability are disproportionately disadvantaged by this requirement, and early spatial difficulties are associated with poorer anatomy performance overall [5]. Virtual Reality (VR) offers a theoretically compelling solution: by presenting anatomical structures within an immersive, stereoscopic environment, VR permits learners to perceive depth, spatial relationships, and structural overlap directly, rather than inferring them from 2D representations. The learner can rotate, dissect, and re-examine a virtual structure indefinitely, facilitating deliberate practice in a way that a physical cadaver cannot [6].

Despite the rapid proliferation of VR tools in medical education, significant scepticism persists regarding their objective benefit over traditional methods. Early studies were frequently criticised for measuring learner satisfaction and novelty-driven enthusiasm rather than durable educational outcomes. Furthermore, the term "VR" has been applied inconsistently across the literature, encompassing fully immersive head-mounted display (HMD) systems, semi-immersive desktop environments, and virtual dissection tables, making cross-study comparison difficult [7]. A meta-analysis by Salimi et al. of 24 RCTs found VR produced a moderate, significant improvement in anatomy knowledge scores compared to conventional methods (SMD = 0.58, p < 0.01); however, the high heterogeneity across pooled studies (I² = 87%) means it remains unclear whether this effect holds consistently across different intervention designs [8].

This systematic review addresses this gap by synthesising the highest available level of evidence: randomised controlled trials (RCTs). By restricting inclusion to RCTs, this review minimises selection bias and provides the most rigorous assessment of whether VR objectively improves knowledge acquisition, long-term retention, and learner satisfaction in undergraduate anatomy education compared to conventional teaching methods.

## Review

Methods

Search Strategy

This review was conducted and reported in accordance with the Preferred Reporting Items for Systematic Reviews and Meta-Analyses (PRISMA) 2020 guidelines [9]. A comprehensive systematic search was performed across PubMed/MEDLINE) and the Cochrane Central Register of Controlled Trials (CENTRAL) from inception to August 2024. No language restrictions were applied. Full text availability was required for inclusion at the eligibility assessment stage.

Search terms combined Medical Subject Headings (MeSH) and free text terms across three domains: (i) intervention: "Virtual Reality," "VR," "head-mounted display," "HMD," "immersive simulation," "virtual dissection"; (ii) subject: "anatomy," "anatomical education," "gross anatomy"; (iii) study design: "randomised controlled trial," "RCT." Boolean operators were used to combine domains.

The PubMed search string was: ("virtual reality"[tiab] OR "immersive VR"[tiab] OR "head-mounted display"[tiab] OR "virtual dissection"[tiab]) AND ("anatomy"[tiab] OR "anatomical education"[tiab] OR "gross anatomy"[tiab]) AND ("randomised controlled trial"[pt] OR "randomized controlled trial"[pt] OR "RCT"[tiab]), yielding 48 records.

The search in CENTRAL was: ("virtual reality" OR "immersive VR" OR "head-mounted display" OR "virtual dissection") AND ("anatomy" OR "anatomical education" OR "gross anatomy"), yielding 178 records.

Eligibility Criteria

Inclusion criteria were: (i) RCT study design; (ii) undergraduate medical student population; (iii) VR intervention defined as an immersive or semi-immersive 3D digital environment (HMD-based or virtual dissection table); (iv) comparator of conventional teaching (cadaveric dissection, 2D textbooks, lectures, or standard online resources); and (v) at least one objective outcome measure (knowledge acquisition via post-test scores, long-term retention via delayed testing post-intervention, or validated learner satisfaction scale).

All included studies employed a randomised controlled design with random allocation to intervention and control conditions and objective outcome measurement. Exclusion criteria were: (i) studies using augmented reality (AR) without a VR arm; (ii) non-immersive 2D computer-based learning alone; (iii) postgraduate or specialist trainees as the primary population; (iv) non-randomised or observational designs (cluster-randomised controlled studies were considered eligible); (v) studies reporting only subjective usability outcomes without objective knowledge assessment; and (vi) studies for which full text was unavailable.

Outcomes

The primary outcome was immediate knowledge acquisition, measured as post-intervention test scores. Secondary outcomes were: long-term knowledge retention; learner satisfaction (measured using validated instruments); and, where reported, cognitive load and cost-effectiveness.

Study Selection and Data Extraction

Two reviewers (RE and IK) independently screened titles and abstracts, followed by full-text review of potentially eligible studies. Disagreements were resolved by discussion and, where necessary, by a third reviewer (SG). Data were extracted using a standardised extraction form capturing: study design, population characteristics, sample size, VR modality, comparator, outcome measures, results, and follow-up duration.

Quality Assessment

Risk of bias was assessed independently by two reviewers (RE and IK) using the Cochrane Risk of Bias 2.0 (RoB 2.0) tool, which evaluates five domains: randomisation process, deviations from intended intervention, missing outcome data, measurement of the outcome, and selection of the reported result [10]. Given the inherent impossibility of blinding participants to educational technology interventions, performance bias was anticipated across studies.

Data Synthesis

Owing to clinical and methodological heterogeneity across included studies, including variation in VR modality, anatomical domain, comparator type, and outcome measurement instruments, a narrative synthesis was performed in accordance with SWiM (Synthesis Without Meta-analysis) reporting guidance [11]. Where studies reported comparable outcome measures, results are summarised descriptively with effect sizes and p-values as reported by the original authors.

Results

Study Selection

**Figure 1:**
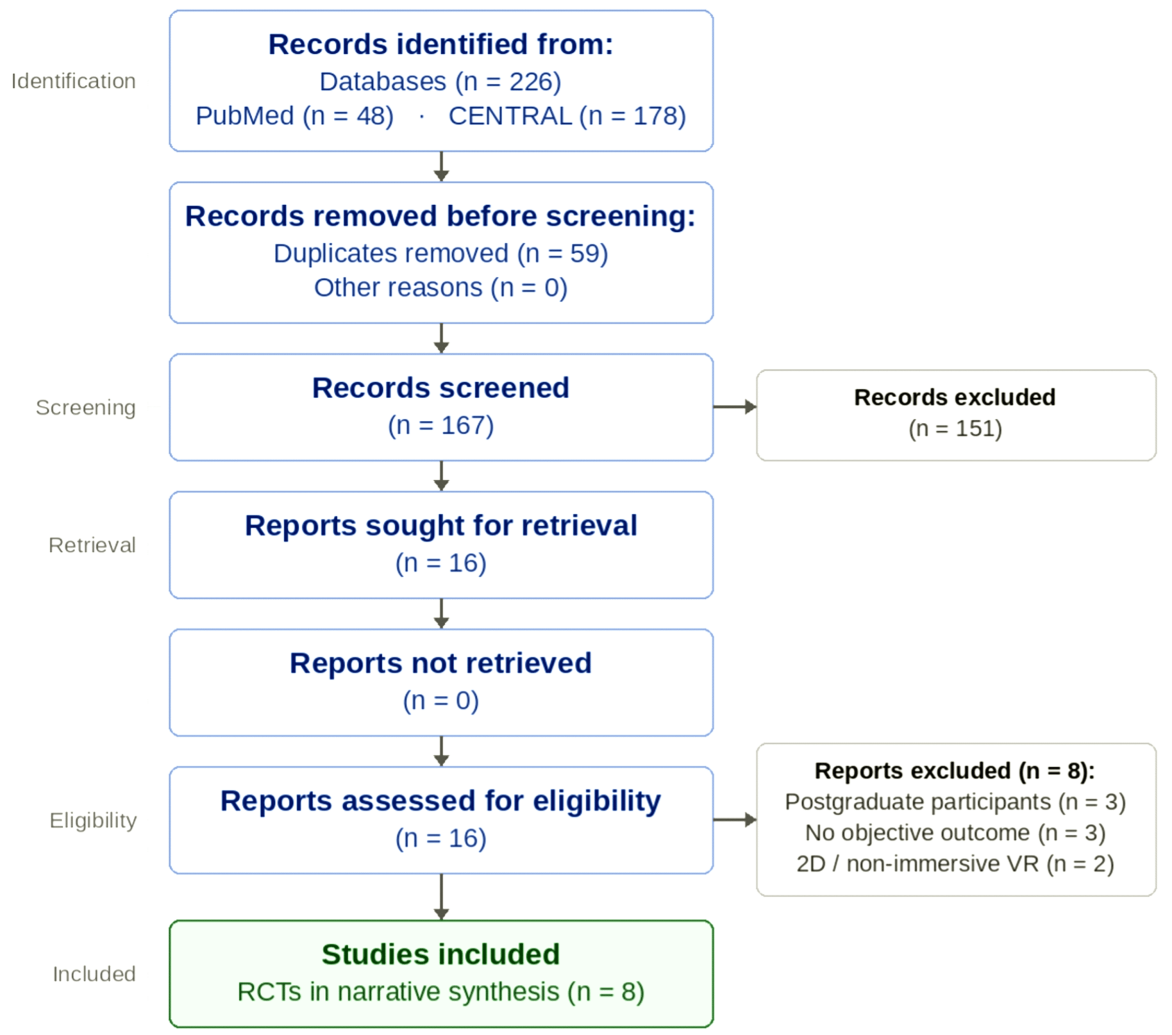
PRISMA flow diagram summarising study selection process PRISMA: Preferred Reporting Items for Systematic Reviews and Meta-Analyses; RCT: randomised controlled trial; VR: virtual reality

The search yielded 226 records in total (48 in PubMed and 178 in CENTRAL). Following deduplication, 167 records were screened by title and abstract, of which 151 were excluded as not meeting inclusion criteria. Sixteen full-text articles were assessed for eligibility, of which eight were excluded: three included postgraduate trainees as the primary participant group, three reported no objective knowledge outcome, and two used 2D-only or non-immersive VR that did not meet the modality inclusion criterion. Eight RCTs, enrolling approximately 480 undergraduate medical students, met all eligibility criteria and were included in this review. The study selection process is summarised in the PRISMA flow diagram (Figure 1).

Characteristics of Included Studies

The eight included RCTs were published between 2004 and 2023 and encompassed a range of anatomical domains and VR modalities. Table 1 provides a structured summary of included study characteristics, interventions, comparators, and key outcomes.

**Table 1:** Characteristics of included randomised controlled trials. RCT: randomised controlled trial; VR: virtual reality; HMD: head-mounted display; IVR: immersive VR

Study (Year)	Study Design	Number of Participants	Anatomical Domain	VR Modality	Comparator	Primary Outcome	Key Result
Koucheki et al. (2023) [12]	Crossover RCT	50	Skeletal anatomy (upper and lower limb)	HMD-based IVR	Cadaveric bone models	Knowledge acquisition	No significant difference between IVR and cadaveric bone groups at either stage (upper limb p = 0.286; lower limb p = 0.936); non-inferiority formally demonstrated; students favoured combination use of IVR and traditional methods
Maresky et al. (2019) [13]	Parallel RCT	42	Cardiac anatomy	Immersive HMD-based VR	2D textbook	Spatial knowledge acquisition	Significantly improved scores in VR group overall (p < 0.001); greatest effect on visual-spatial content (26.4% higher, p < 0.001) versus conventional content (21.4% higher, p = 0.004)
Deng et al. (2018) [14]	Parallel RCT	120	Gross anatomy	Virtual dissection table (anatomage-style)	Traditional cadaveric dissection	Knowledge acquisition and failure rate	VR group significantly higher gross anatomy exam scores (p < 0.01); marked reduction in failure rates
Ebert et al. (2019) [15]	Parallel RCT	51	Fetal anatomy (ultrasound)	Semi-immersive 3D VR (interactive), as supplement to traditional lecture	Traditional lecture alone	Knowledge retention (4 months)	Superior retention at 4 months (p < 0.001); interactivity identified as key mechanism
Ekstrand et al. (2018) [16]	Parallel RCT	64	Neuroanatomy	HMD-based immersive VR (HTC Vive)	Paper-based textbook	Knowledge acquisition and 7-day retention	Equivalent post-test and 7-day retention scores; significant improvement from baseline in both groups; VR group reported higher satisfaction and reduced neurophobia
Stepan et al. (2017) [17]	Parallel RCT	66	Neuroanatomy	HMD-based IVR	Online textbook / 2D resources	Knowledge acquisition, 8-week retention and motivation	Equivalent test scores at post-test and 8-week retention; VR group significantly higher motivation (p < 0.01)
Nicholson et al. (2006) [18]	Parallel RCT	57	Ear / temporal bone	3D virtual model (desktop), as supplement to web-based tutorial	Web-based tutorial alone	Knowledge acquisition	VR cohort significantly outperformed control (p < 0.001); largest effect in spatial orientation items
Hariri et al. (2004) [19]	Parallel RCT	29	Shoulder / arthroscopic	Semi-immersive VR (desktop)	Printed textbook	Knowledge acquisition	Equivalent post-test scores (p = 0.70); VR group significantly higher satisfaction

Risk of Bias

**Figure 2:**
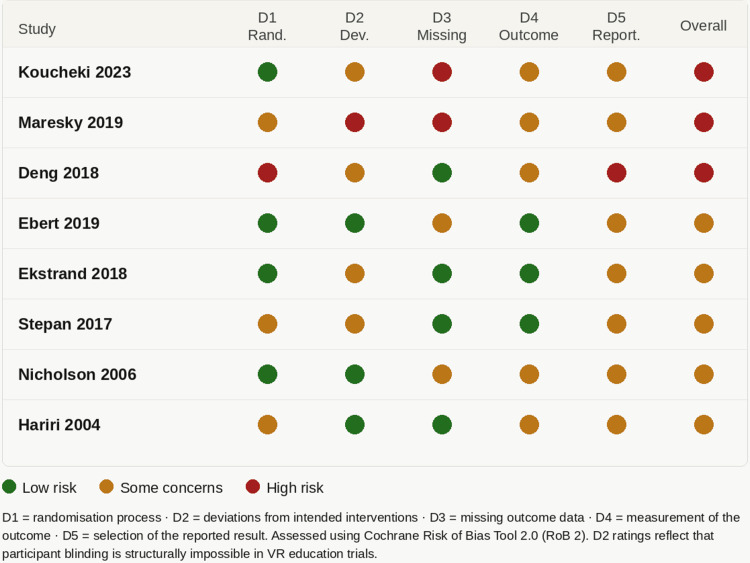
Risk of bias tool 2.0 (RoB 2) Studies shown: Koucheki et al. [12], Maresky et al. [13], Deng et al. [14], Ebert et al. [15], Ekstrand et al. [16], Stepan et al. [17], Nicholson et al. [18], Hariri et al. [19]

Risk of bias was assessed using the Cochrane Risk of Bias Tool 2.0 across five domains (Figure 2) [10]. As blinding of participants is not possible in VR education trials, Domain 2 ratings were based on whether actual protocol deviations occurred rather than blinding status alone. Three studies were rated as having a high overall risk of bias [12-14], and five were rated as having some concerns [15-19]. The most consistent weakness across all studies was the lack of prospective registration. High-risk ratings were driven primarily by poor handling of participant dropout and, in Deng et al. [14], allocation at the class rather than the individual level. No study achieved a low overall risk rating.

Knowledge Acquisition

All eight included RCTs assessed post-intervention knowledge acquisition as a primary or co-primary outcome. In three [13,14,18] of these eight trials, VR was associated with statistically superior performance compared to the control condition. The most pronounced effects were observed in domains characterised by high spatial complexity. Nicholson et al. reported that students whose web-based tutorial included an interactive 3D virtual model of the temporal bone significantly outperformed those who completed the same tutorial without the model (p < 0.001), with the largest between-group differences seen on items assessing three-dimensional spatial orientation of inner ear structures [18]. Similarly, Maresky et al. demonstrated that immersive VR produced significantly greater gains in cardiac anatomy knowledge compared to 2D textbook study, with the largest effect on visual-spatial content (26.4% higher, p < 0.001) and a significant effect on conventional content (21.4% higher, p = 0.004) [13]. Deng et al. reported significantly higher gross anatomy examination scores in students using a virtual dissection table compared to cadaveric dissection alone (p < 0.01) [14]. In contrast, Stepan et al. [17], Hariri et al. [19], Ekstrand et al. [16], and Koucheki et al. [12] reported equivalent or non-inferior knowledge scores between VR and control groups. Koucheki et al. conducted the only crossover non-inferiority RCT in the review, randomising first-year medical students to HMD-based VR or cadaveric bone models for skeletal anatomy; no significant difference was found at any stage, and non-inferiority was formally demonstrated [12]. Notably, Ekstrand et al. administered a retention test at five to nine days post intervention and found no significant difference between the immersive VR and paper-based groups at either time point, though both groups showed significant improvement from baseline [16]. These null-difference findings are collectively informative: they indicate that VR is at least non-inferior to conventional methods, supporting its use as an acceptable alternative where traditional resources are constrained.

Long-Term Knowledge Retention

Knowledge retention beyond the immediate post-test was assessed in three of the eight included RCTs. Across these studies, the consistent pattern is one of non-inferiority at shorter follow-up intervals and a possible advantage at longer intervals. Stepan et al. administered a retention quiz at eight weeks post intervention and found no significant difference between the VR and online textbook groups, confirming that VR-acquired knowledge is durably maintained at least as well as conventionally acquired knowledge over an extended period [17]. Ekstrand et al. similarly found no significant difference at five to nine days between immersive VR (IVR) and a paper-based study, with both groups maintaining the gains seen immediately post intervention [16]. Ebert et al. demonstrated superior knowledge retention at four months in students who received traditional lectures supplemented with an interactive 3D VR model, compared to those who received traditional lectures alone (p < 0.001) [15]. This study is notable for its identification of interactivity as a potentially more important mediator of retention than full immersion per se; the ability to manipulate and re-examine a structure repeatedly was proposed as the mechanism underlying durable memory encoding. Taken together, these three studies provide convergent evidence that VR is at minimum non-inferior to conventional methods for long-term knowledge retention, with a single but methodologically sound signal of superiority at four months. Follow-up assessments extending beyond four months remain absent from the literature and represent a priority for future research.

Learner Satisfaction and Engagement

Learner satisfaction was reported across four studies and consistently favoured VR. Hariri et al. reported significantly higher satisfaction in the VR group despite equivalent knowledge acquisition, suggesting that VR confers motivational benefits independent of its direct cognitive effects [19]. Stepan et al. found significantly greater self-reported motivation in the VR group (p < 0.01), which the authors hypothesised may translate to improved long-term study habits even when short-term test performance is equivalent [17]. Ekstrand et al. reported that the VR group demonstrated significantly reduced neurophobia (a well-documented reluctance to engage with neuroanatomy) alongside higher satisfaction ratings compared to the paper-based group, despite equivalent objective performance [16]. This finding is particularly noteworthy as it suggests VR may confer meaningful affective benefits in anatomical domains associated with learner anxiety, independent of direct effects on knowledge scores. Koucheki et al. additionally reported high learner acceptance of IVR, with 79% of students identifying it as most valuable for teaching 3D orientation and anatomical relationships, and a majority expressing a preference for the combination use of IVR and traditional methods over either modality alone [12].

Discussion

The Spatial Advantage: Cognitive Load Theory and VR

The most consistent finding across this review is that VR confers its greatest benefits in anatomical domains characterised by high spatial complexity. This observation is directly predicted by cognitive load theory (CLT), originally formulated by Sweller [20] and later elaborated by Paas and colleagues [21]. Within CLT, extraneous cognitive load refers to mental effort imposed by the manner in which information is presented, rather than by the intrinsic difficulty of the material itself. When students attempt to form 3D mental models from 2D images, the resulting extraneous load consumes working memory resources that would otherwise be available for germane processing, the meaningful integration of new knowledge into existing schemas. VR reduces extraneous load by presenting 3D structures directly and perceptually, effectively outsourcing the mental rotation task to the technology [4].

This theoretical framework now has direct physiological support. A study by Lowry et al., although not one of the included RCTs in this review, provided the first biometric evidence of VR’s effect on cognitive load in anatomy education by demonstrating, via real-time biometric measurement including pupillometry, that students viewing anatomy in a 3D immersive VR environment exhibited significantly lower cognitive load than those studying identical structures on a 2D screen [22]. This objective physiological finding corroborates what CLT predicts theoretically and offers a mechanistic explanation for VR’s learning advantages that go beyond self-report measures.

This CLT framework explains the differential effects observed across studies. Studies by Nicholson et al. [18] and Maresky et al. [13], which targeted regions of particularly high spatial complexity (temporal bone, cardiac anatomy), demonstrated statistically significant superiority of VR, consistent with CLT’s prediction that extraneous load reduction benefits are greatest where 2D representation is most inadequate. In contrast, studies by Hariri et al. (arthroscopic shoulder) [19] and Koucheki et al. (skeletal anatomy versus cadaveric bone) [12] showed non-inferior but not superior performance, reflecting domains where physical models already provide relatively direct 3D experience. The CLT lens also accounts for the "levelling" effect observed by Deng et al. [14], whereby the virtual dissection table was associated with the elimination of the 8.6% failure rate seen in the control group and a rise in high scores (>85) from 51.46% to 61.23%; students with lower baseline spatial ability are theoretically most likely to benefit from VR's extraneous load reduction [4], though Deng et al. did not formally examine differential effects by ability subgroup [14].

Long-Term Retention: Deliberate Practice and the Testing Effect

This review identified three RCTs that addressed retention, revealing a coherent pattern: VR is non-inferior to conventional methods at shorter follow-up intervals (eight weeks [17], five to nine days [16]), and a single methodologically sound RCT [15] demonstrates superiority at four months (p < 0.001). The non-inferiority findings are themselves important, confirming that knowledge acquired via VR is retained at least as well as conventionally acquired knowledge and addressing a plausible concern that learning in novel technology environments may be more superficial or temporary. The proposed mechanism underlying Ebert et al.'s superiority centres on VR's unique repeatability. It should be noted, however, that Ebert et al. [15] employed an add-on design in which both groups received traditional teaching; the possibility that additional learning time rather than VR itself drove the retention advantage cannot be fully excluded. Unlike a cadaveric specimen, which is dissected once and thereafter progressively degraded, a virtual model can be reset and re-examined an unlimited number of times. This characteristic enables two well-evidenced pedagogical strategies: deliberate practice [23] and spaced retrieval [24]; both of which are robustly associated with long-term memory consolidation. Future research should examine whether this retention advantage persists beyond four months and whether it is moderated by the frequency of repeated VR practice sessions.

VR as Adjuvant: The Blended Learning Imperative

A recurring and important theme in this evidence base concerns what the RCT findings can and cannot be taken to mean for curriculum design. Each included RCT compared VR to one specific traditional element, for example, a textbook module, a lecture series, or a cadaveric session, for a defined anatomical topic. This is precisely the correct experimental question: whether VR can perform at least as well as, or better than, a conventional teaching method in a head-to-head comparison. The findings presented in this review demonstrate that it can. However, it does not follow that VR should wholesale replace every element of the anatomy curriculum. Cadaveric dissection provides qualities that current VR cannot replicate: genuine haptic feedback, appreciation of biological variation between individuals, and the professional and psychosocial dimensions of handling human remains, experiences that have been argued to contribute to the development of professional identity in medical students [25,26].

The weight of this evidence therefore supports a blended learning model, in which VR is used for initial spatial orientation and repeated review, while cadaveric dissection provides subsequent tactile experience and contextualisation of biological variability. This model is consistent with constructivist learning theory, which holds that learners build new knowledge on existing scaffolding: VR provides the cognitive scaffold of 3D orientation, upon which the richer, more complex experience of dissection can be layered [26]. Pragmatically, this blended approach also optimises the use of increasingly scarce cadaveric resources.

Cost-Effectiveness and Sustainability

An important dimension of the case for VR adoption that extends beyond pure educational efficacy is economic sustainability. Although none of the included RCTs conducted a formal cost-effectiveness analysis that met this review’s quality standards, the structural cost advantages of VR over cadaveric laboratory provision are well documented in the broader literature. In the context of expanding medical school cohorts, tightening institutional budgets, and the post-pandemic acceleration of digital education infrastructure, the scalability of VR is a compelling advantage: a single high-quality virtual model can be accessed simultaneously by hundreds of students, without the physical constraints of laboratory space or the logistical requirements of cadaver procurement. For medical schools in low- and middle-income countries, where cadaveric access is particularly limited, VR may represent a genuinely transformative educational technology rather than merely a supplementary one [27].

Limitations of This Review

Several limitations of the available evidence base must be acknowledged. First, performance bias is the predominant methodological concern across included studies. Because participants inevitably know which educational technology they are receiving, motivational differences between groups, the so-called novelty effect, cannot be fully eliminated. Researchers are encouraged to include acclimatisation periods in future trials and to use blinded assessors for all objective outcome measurements. Second, all included studies are single-centre, with relatively small sample sizes ranging from 29 to 120 participants, limiting generalisability. Multi-centre RCTs across diverse institutional contexts are needed. Third, there is considerable heterogeneity in what is classified as "VR" across studies. The spectrum from fully immersive HMD-based environments to semi-immersive virtual dissection tables encompasses substantially different experiential qualities and hardware requirements. Future research should adopt standardised terminology and explicitly report VR immersion level to enable more meaningful comparison.

Finally, this review does not address the question of whether VR is equally effective across all student subgroups. While CLT would predict greater gains in students with lower baseline spatial ability, no included study formally examined effect modification by prior spatial ability, learning style, or familiarity with digital technology. These represent important priorities for future research. Additionally, two included studies [15,18] used an add-on design in which both groups received conventional teaching, and the intervention group received VR in addition; this limits direct comparability with the remaining six studies and means that additional learning time cannot be fully excluded as a contributing factor to the superior outcomes observed in those trials. Unlike larger meta-analyses that pool many different VR interventions into a single effect size, this review examined each study individually. A 2024 meta-analysis found VR and AR produced greater knowledge gains than traditional teaching overall (SMD = 0.40), with the largest effect when VR supplemented rather than replaced conventional teaching [28]. Four of our eight included studies reported statistically significant improvements in knowledge outcomes: two used add-on designs [15,18], and two used direct substitution designs [13,14], indicating that a positive result in this literature is not confined to either approach.

## Conclusions

This systematic review of eight RCTs demonstrates that VR is an effective and evidence-based tool for undergraduate anatomy education, consistently matching or exceeding conventional teaching methods in immediate knowledge acquisition and long-term knowledge retention. Its primary mechanisms of benefit are the reduction of extraneous cognitive load through direct 3D perception and the unique repeatability of virtual models, which enables deliberate practice and spaced retrieval. Critically, where VR did not outperform conventional methods, it was consistently non-inferior, supporting its use as an acceptable alternative in settings where traditional resources are constrained. The evidence therefore supports integration of VR within a blended learning model, complementing rather than replacing cadaveric dissection, which continues to provide haptic feedback, exposure to biological variation, and the professional formation experiences that current VR cannot replicate.

Given the scalability and sustainability advantages of VR, particularly its potential to widen access in institutions where cadaveric provision is limited, its incorporation into modern anatomy curricula appears both educationally justified and practically compelling. Future research should prioritise multi-centre trials with retention follow-up beyond four months, adoption of a standardised VR taxonomy to enable meaningful cross-study comparison, formal cost-effectiveness analyses, and investigation of differential benefits across student subgroups, particularly those with lower baseline spatial ability.

## References

[1] Drake RL, McBride JM, Pawlina W (2014). An update on the status of anatomical sciences education in United States medical schools. Anat Sci Educ.

[2] Older J (20041). Anatomy: a must for teaching the next generation. Surgeon.

[3] Patel KM, Moxham BJ (2006). Attitudes of professional anatomists to curricular change. Clin Anat.

[4] Sweller J (1994). Cognitive load theory, learning difficulty, and instructional design. Learn Instruct.

[5] Guillot A, Champely S, Batier C, Thiriet P, Collet C (2007). Relationship between spatial abilities, mental rotation and functional anatomy learning. Adv Health Sci Educ Theory Pract.

[6] Mason L, Holmes M (2019). Virtual reality as a teaching aid for anatomy. INTED2019 Proceedings.

[7] Hackett M, Proctor M (2016). Three-dimensional display technologies for anatomical education: a literature review. J Sci Educ Tech.

[8] Salimi S, Asgari Z, Mohammadnejad A, Teimazi A, Bakhtiari M (2024). Efficacy of virtual reality and augmented reality in anatomy education: a systematic review and meta-analysis. Anat Sci Educ.

[9] Page MJ, McKenzie JE, Bossuyt PM (2021). The PRISMA 2020 statement: an updated guideline for reporting systematic reviews. BMJ.

[10] Sterne JA, Savović J, Page MJ (2019). RoB 2: a revised tool for assessing risk of bias in randomised trials. BMJ.

[11] Campbell M, McKenzie JE, Sowden A (2020). Synthesis without meta-analysis (SWiM) in systematic reviews: reporting guideline. BMJ.

[12] Koucheki R, Lex JR, Morozova A (2023). Immersive virtual reality and cadaveric bone are equally effective in skeletal anatomy education: a randomized crossover noninferiority trial. J Surg Educ.

[13] Maresky HS, Oikonomou A, Ali I, Ditkofsky N, Pakkal M, Ballyk B (2019). Virtual reality and cardiac anatomy: exploring immersive three-dimensional cardiac imaging, a pilot study in undergraduate medical anatomy education. Clin Anat.

[14] Deng X, Zhou G, Xiao B, Zhao Z, He Y, Chen C (2018). Effectiveness evaluation of digital virtual simulation application in teaching of gross anatomy. Ann Anat.

[15] Ebert J, Tutschek B (2019). Virtual reality objects improve learning efficiency and retention of diagnostic ability in fetal ultrasound. Ultrasound Obstet Gynecol.

[16] Ekstrand C, Jamal A, Nguyen R, Kudryk A, Mann J, Mendez I (2018). Immersive and interactive virtual reality to improve learning and retention of neuroanatomy in medical students: a randomized controlled study. CMAJ Open.

[17] Stepan K, Zeiger J, Hanchuk S, Del Signore A, Shrivastava R, Govindaraj S, Iloreta A (2017). Immersive virtual reality as a teaching tool for neuroanatomy. Int Forum Allergy Rhinol.

[18] Nicholson DT, Chalk C, Funnell WR, Daniel SJ (2006). Can virtual reality improve anatomy education? A randomised controlled study of a computer-generated three-dimensional anatomical ear model. Med Educ.

[19] Hariri S, Rawn C, Srivastava S, Youngblood P, Ladd A (2004). Evaluation of a surgical simulator for learning clinical anatomy. Med Educ.

[20] Sweller J, Van Merrienboer JJ, Paas FG (1998). Cognitive architecture and instructional design. Educ Psychol Rev.

[21] Paas F, Renkl A, Sweller J (2003). Cognitive load theory and instructional design: recent developments. Educ Psychol.

[22] Lowry B, McGrath S, Eitel C (2026). Cognitive load in virtual reality anatomy education: comparing 2D and 3D learning experiences. Med Sci Educ.

[23] Ericsson KA, Krampe RT, Tesch-Römer C (1993). The role of deliberate practice in the acquisition of expert performance. Psychol Rev.

[24] Roediger HL, Karpicke JD (2006). Test-enhanced learning: taking memory tests improves long-term retention. Psychol Sci.

[25] Hafferty FW (1991). Into the Valley: Death and the Socialization of Medical Students. https://search.worldcat.org/title/1027752328.

[26] Vygotsky LS (1978). Mind in Society : The Development of Higher Psychological Processes. https://archive.org/details/mindinsocietydev00vygo.

[27] Haerling KA (2018). Cost-utility analysis of virtual and mannequin-based simulation. Simul Healthc.

[28] García-Robles P, Cortés-Pérez I, Nieto-Escámez FA, García-López H, Obrero-Gaitán E, Osuna-Pérez MC (2024). Immersive virtual reality and augmented reality in anatomy education: a systematic review and meta-analysis. Anat Sci Educ.

